# Olfactory testing as COVID-19 screening in school children; A prospective cross-sectional study

**DOI:** 10.1371/journal.pone.0277882

**Published:** 2022-11-22

**Authors:** Sarah A. Gitomer, Kaitlyn Tholen, Kaci Pickett, Rakesh D. Mistry, Daniel M. Beswick, Jill L. Kaar, Brian W. Herrmann

**Affiliations:** 1 Division of Pediatric Otolaryngology, Children’s Hospital Colorado, Aurora, CO, United States of America; 2 Department of Otolaryngology–Head and Neck Surgery, University of Colorado School of Medicine, Aurora, CO, United States of America; 3 The Center for Research in Outcomes for Children’s Surgery, University of Colorado School of Medicine, Aurora, CO, United States of America; 4 Section of Emergency Medicine, Children’s Hospital Colorado, Aurora, CO, United States of America; University Putra Malaysia, MALAYSIA

## Abstract

**Background:**

Little is known about olfactory changes in pediatric COVID-19. It is possible that children under-report chemosensory changes on questionnaires, similar to reports in adults. Here, we aim to describe COVID-19-related olfactory dysfunction in outpatient children. We hypothesized that children with COVID-19 will demonstrate abnormal olfaction on smell-identification testing at a higher rate than children with negative COVID-19 testing.

**Methods:**

A prospective cross-sectional study was undertaken from June 2020—June 2021 at a tertiary care pediatric hospital. A consecutive sample of 205 outpatients aged 5–21 years undergoing severe acute respiratory syndrome coronavirus 2 (SARS-CoV2) PCR testing were approached for this study. Patients with prior olfactory dysfunction were excluded. Participants were given a standard COVID-19 symptom questionnaire, a Smell Identification Test (SIT) and home-odorant-based testing within 2 weeks of COVID-19 testing. Prior to study enrollment, power calculation estimated 42 patients to determine difference in rates of SIT results between groups. Data were summarized with descriptive statistics.

**Results:**

Fifty-one patients underwent smell identification testing (23 positive (45%) and 28 negative (55%) for COVID-19; mean age 12.7 years; 60% female). 92% of all patients denied subjective change in their sense of smell or taste but only 58.8% were normosmic on testing. There was no difference in screening questionnaires or SIT scores between COVID-19 positive and negative groups.

**Conclusions:**

Unlike adults, there was no statistical difference in olfactory function between outpatient COVID-19 positive and negative children. Our findings suggest a discrepancy between objective and patient-reported olfactory function in pediatric patients, and poor performance of current screening protocols at detecting pediatric COVID-19.

## Introduction

Little has been documented about impact of coronavirus disease 2019 (COVID-19) on olfaction in outpatient children. Early in the pandemic, prior to widespread availability of rapid testing screening for COVID-19, there were numerous challenges in developing an optimal screening protocol. One major limitation was that symptomatic variability may mask early SARS-CoV2 infection, and COVID-19 symptoms often mimic other common respiratory infections [1]. However, with early variants of SARS-CoV2, olfactory dysfunction was much more common in adult COVID-19 than in other viral illnesses [2–4] and there were high rates of disturbances in chemosensory perception in adults with early SARS-CoV2 infection [5–8]. It has been reported that changes in olfaction can be detected immediately after confirmation of COVID-19 in hospitalized children [9]. We hypothesized that chemosensory screening would detect olfactory changes in outpatient children with early COVID-19 at a higher rate than outpatient children without COVID-19. If this were true, it could be possible to develop a simple chemosensory-based screening test to detect early infection. Based on modelling studies, in combination with molecular testing, self-administered olfactory testing has been proposed as a high-impact, cost-effective tool for screening for COVID-19 [10]. For instance, if a simple, inexpensive olfactory test with good specificity were administered weekly to the US population (with reflex molecular testing), this would represent a 40X-200X less expensive screening tool than weekly antigen or PCR testing [10]. The first step toward creating such a test is to identify if measured olfactory changes vary between COVID-19 positive and negative children, using more complex, validated testing.

In general, children report less severe symptoms of acute COVID-19 infection [11–14]. Small observational studies of children have noted a lower rate of reported changes in taste and smell [11, 15], but it is unknown if children truly have a lower rate of these symptoms, or are not able to subjectively report dysgeusia or dysosmia. There is currently no study comparing outpatient SARS-CoV2 positive pediatric patients to SARS-CoV2 negative controls in order to address this knowledge gap.

Standardized chemosensory testing is complementary to patient-reported olfactory changes in adults [16–19] and may be vital for pediatrics as young children may not articulate changes in smell without direct testing. The objectives of this study were to describe chemosensory changes in asymptomatic children and adolescents with COVID-19, and to evaluate if objective testing of odor identification via the Smell Identification Test (SIT) was associated with results of SARS-CoV2 testing. We hypothesized that asymptomatic children with COVID-19 would have higher rates of microsmia and anosmia as identified with olfactory testing than children without COVID-19.

### Aim

This study aims to describe olfactory dysfunction in outpatient pediatric COVID-19 using a standardized, validated SIT. Secondary aims included evaluating rates of olfactory dysfunction in children with COVID-19 in comparison to children without disease.

## Materials and methods

### Study design and setting

This was a prospective cross-sectional study conducted early in the COVID-19 pandemic at a single center: the main campus of a tertiary care pediatric institution. Children’s Hospital of Colorado is a 434-bed pediatric hospital located in Aurora, Colorado and serves a seven-state catchment area in the western United States.

### Participants

The project was approved by the Colorado Multiple Institutional Review Board (COMIRB). Children aged 5–20 years undergoing SARS-CoV2 PCR testing from June 2020 to June 2021 were approached for inclusion. Written short-form consent was obtained from patients or from parents/guardians of minors. COMIRB waived full-form written informed consent. Recruitment was prior to Delta and Omicron variant waves. Patients were included if they were undergoing SARS-CoV2 testing as outpatients for pre-operative screening prior to elective surgeries or in the emergency department for pre-admission screening prior to hospitalization for non-respiratory complaints (i.e. after extremity trauma). Patients with prior smell/taste disturbance, severe food allergies, acute altered mental status or who were unable to complete the smell testing assessments were excluded.

### Data collection

For each consenting participant, the following demographic information were recorded: age, sex, race, ethnicity, and date of SARS-CoV2 PCR testing. Patients were given iPads to complete surveys and were either instructed in person (COVID negative patients) or over the telephone with additional instruction via email (COVID positive patients and patients under investigation) about how to complete the SIT. Participants completed the hospital-standard COVID-19 symptom screening questionnaire. They were additionally administered the 40-Question SIT [20], a validated measure of anosmia, to be completed within 2 weeks of PCR testing. The University of Pennsylvania SIT (UPSIT) was chosen because it has been validated in young children, it has a wide variety of scents, and is a single-use test (which was important for use in COVID-19 patients). This SIT is validated in children as young as 5-years-old and consists of 40 scratch-and-sniff scents accompanied by a multiple-choice question for each scent, and it categorizes patients’ smell as normal or abnormal compared to age- and sex-matched controls. Social distancing protocols limited our ability to recruit patients face-to-face in the hospital or outpatient settings, and so the delivery and completion of the SIT within 2 weeks of laboratory testing was accomplished via remote methods (mailing SIT tests) and providing clear instructions to participants. These directions were given over the phone, with a written copy of instructions provided via email. Additional testing was completed at home for all age groups, based on the SmellTracker protocol from the Weizmann Olfaction Group [21]. Parents were instructed to have the participants (children) close their eyes and try to identify household items such as toothpaste, peanut butter, vinegar, vanilla extract, and garlic. All study data were collected and managed using REDCap electronic data capture tools hosted at the University of Colorado Anschutz Medical Campus [22].

### Statistical analysis

#### Sample size

Prior to initiating data collection, sample size was calculated to determine how many patients to include to achieve sufficient power to detect differences in SIT. Assuming that 20% of the healthy population have hyposmia or anosmia [23, 24] and predicting that 61% of children with COVID-19 would suffer from hyposmia or anosmia (based on the previously available literature studying olfaction in adult populations [25, 26]) the number of patients to recruit was calculated prior to enrollment. It was estimated that 42 patients (21 SARS-CoV2 positive and 21 SARS-CoV2 negative) would be required in order to obtain power of 0.8, with two-sided alpha 0.05, to show a difference in rates of abnormal SIT results between the two groups.

#### Data analysis

Descriptive statistics were summarized for continuous variables with medians and interquartile ranges, and for categorical variables with frequencies and proportions. Group differences are tested via t-test or Kruskal-Wallis test for continuous variables and Chi Squared test or Fisher’s Exact tests for categorical variables.

## Results

**Fig 1:**
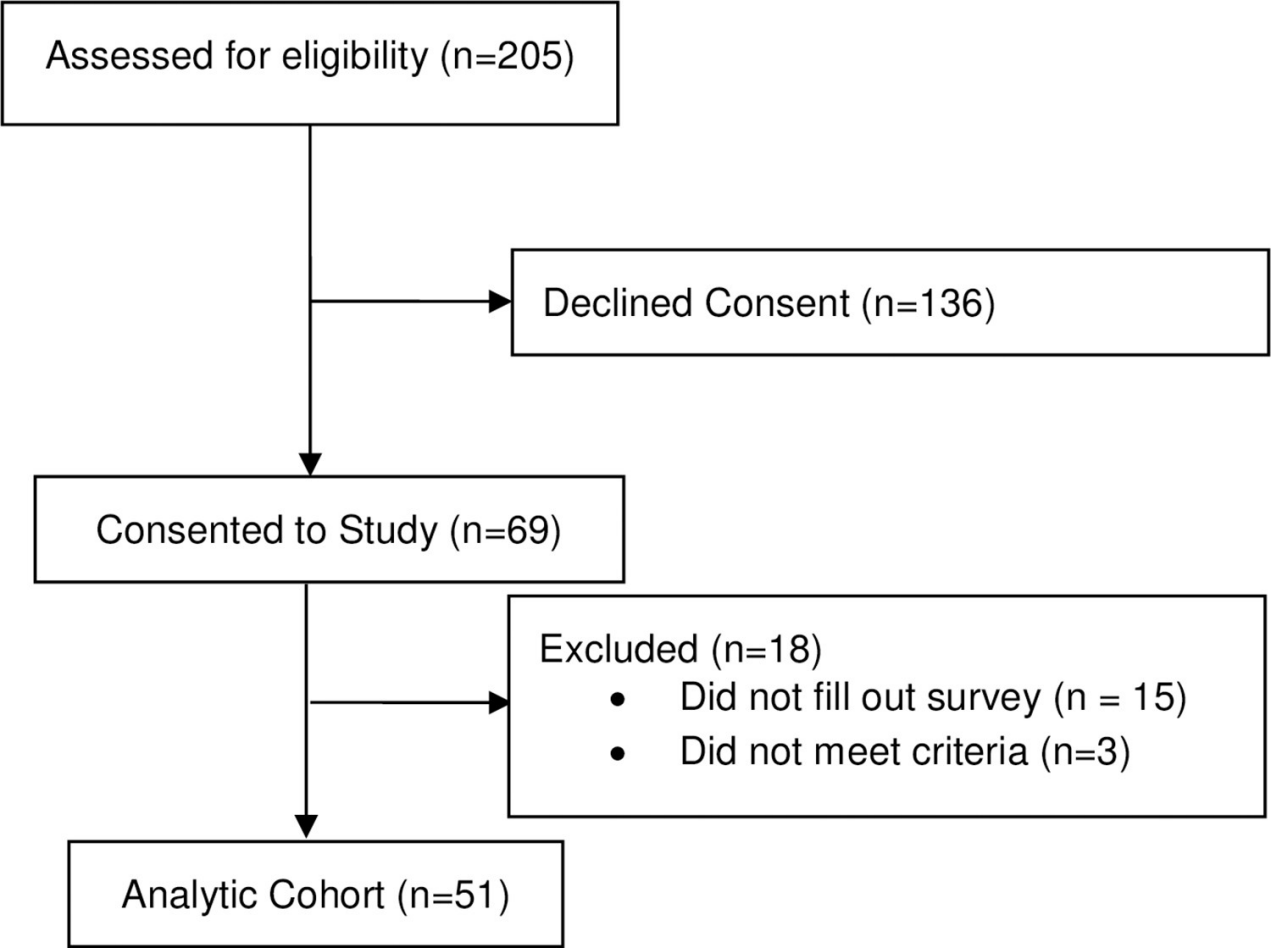
CONSORT diagram.

Prior to data collection, sample size needed to obtain power of 0.8 to detect a difference in rate of anosmia between SARS-CoV2 positive and negative groups was calculated to be 42 patients. A total of 205 patients met study criteria and were approached for the study; 69 consented to participate. Of these, 51 started and 50 completed the chemosensory testing (CONSORT diagram presented in Fig 1). Demographics of the study population are presented in Table 1. Twenty-three (45%) patients tested positive for COVID-19 and 28 (55%) tested negative. There was no significant difference in terms of age, ethnicity, race, or sex between COVID-19 positive and negative subjects. All enrolled patients completed the standard COVID-19 screening questionnaire used at our institution (Table 2); one subject did not complete the entire SIT questionnaire; incomplete answers were excluded from analysis and overall score. There was no difference in any of the reported 10 symptoms between the COVID-19 positive and negative groups, including report of subjective changes in sense of taste or smell. Overall, there was a low rate of subjective chemosensory changes in this cohort. Of the 51 patients, only 4 children (8%) reported a change in sense of smell and 3 children (6%) reported a change in sense of taste. Subjective taste and/or smell disturbances were reported in 3.7% (1/28) of COVID-19 negative, and 13% (3/23) of COVID-19 positive patients, there was no significant difference between the 2 groups (Table 2).

**Table 1:** Demographics of 51 children who underwent COVID-19 testing.

	Positive (N = 23)	Negative (N = 28)	Total (N = 51)	p value
**Age**				0.925
Mean (SD)	12.93 (3.62)	12.57 (4.83)	12.73 (4.29)	
Median (Q1,Q3)	13.08 (10.72, 14.86)	13.34 (7.74, 16.58)	13.31 (9.97, 15.71)	
**Sex**				0.774
Unknown	0	1	1	
Male	10 (43.5%)	10 (37.0%)	20 (40.0%)	
Female	13 (56.5%)	17 (63.0%)	30 (60.0%)	
**Race**				0.450
White	18 (78.3%)	16 (57.1%)	34 (66.7%)	
Black	0 (0.0%)	3 (10.7%)	3 (5.9%)	
Asian	1 (4.3%)	3 (10.7%)	4 (7.8%)	
Other	3 (13.0%)	5 (17.9%)	8 (15.7%)	
Unknown	1 (4.3%)	1 (3.6%)	2 (3.9%)	
**Ethnicity**				0.516
Unknown	2	1	3	
Hispanic or Latino	7 (33.3%)	6 (22.2%)	13 (27.1%)	
Not Hispanic or Latino	14 (66.7%)	21 (77.8%)	35 (72.9%)	

**Table 2:** Standard COVID screening questionnaire from our institution: In the past 14 days have you experienced any of the following?

Current Symptoms	Positive (N = 23)	Negative (N = 28)	Total (N = 51)	p value
Cough	3 (13.0%)	4 (14.8%)	7 (14.0%)	1
Shortness of Breath	3 (13.0%)	5 (18.5%)	8 (16.0%)	0.711
Fever	4 (17.4%)	6 (22.2%)	10 (20.0%)	0.736
Chills	4 (17.4%)	2 (7.4%)	6 (12.0%)	0.395
Muscle Pain	4 (17.4%)	5 (18.5%)	9 (18.0%)	1
Sore Throat	5 (21.7%)	4 (14.8%)	9 (18.0%)	0.715
Congestion	5 (21.7%)	3 (11.1%)	8 (16.0%)	0.444
Runny Nose	3 (13.0%)	4 (14.8%)	7 (14.0%)	1
**Change in sense of smell**	**3 (13.0%)**	**1 (3.7%)**	**4 (8.0%)**	**0.322**
**Change in sense of taste**	**2 (8.7%)**	**1 (3.7%)**	**3 (6.0%)**	**0.588**

Despite a low reported rate (8%) of chemosensory changes in the overall cohort, only 30 children (58.8%) had SIT results consistent with normosmia (Table 3). Children’s overall scores on SIT were not associated with results of SARS-CoV2 testing. There was no significant difference in degree of olfactory changes or the absolute scores on SIT between the COVID-19 positive and negative groups (Table 3). Additionally, there was no significant difference between the SARS-CoV2 positive patients and negative patients on any answer of the SIT.

**Table 3:** SIT scores.

	Positive (N = 23)	Negative (N = 28)	Total (N = 51)	p value
**SIT Total Score**				
Mean (SD)	28.8 (6.36)	30.2 (7.62)	29.5 (7.05)	0.239
**Percentiles**				0.338
Mean (SD)	23.04 (22.57)	26.09 (21.27)	24.72 (21.70)	
Range	3.5–76.00	3.5–98.00	3.5–98.00	
**Category n (%)**				0.096
Normosmia	10 (43.5)	20 (71.4)	30 (58.8)	
Mild microsmia	7 (30.4)	5 (17.9)	12 (23.5)	
Moderate microsmia	2 (8.7)	0 (0)	2 (3.9)	
Severe microsmia	2 (8.7)	0 (0)	2 (3.9)	
Anosmia	2 (8.7)	3 (10.7)	5 (9.8)	

Percentiles and olfactory function were calculated based on sex- and age-matched normative data.

SD = standard deviation.

Finally, on simpler, home-based SIT, there was no significant difference in correct answers in the SARS-CoV2 positive and negative groups. A majority of all children were able to correctly identify the household odorants, and at similar rates in the two groups (Table 4).

**Table 4:** Home odorant olfactory testing results.

	Positive (N = 23) (%)	Negative (N = 22) (%)	Total (N = 45)	p value
**Toothpaste**				0.780
Yes	17 (73.9)	17 (77.3)	34 (75.6)	
No	4 (17.4)	2 (9.1)	6 (13.3)	
Did not test	2 (8.7)	3 (13.6)	5 (11.1)	
**Vinegar**				1
Yes	18 (78.3)	17 (77.3)	35 (77.8)	
No	3 (13.0)	2 (9.1)	5 (11.1)	
Did not test	2 (8.7)	3 (13.6)	5 (11.1)	
**Garlic**				0.637
Yes	13 (56.5)	15 (68.2)	28 (62.2)	
No	6 (26.1)	3 (13.6)	9 (20.0)	
Did not test	4 (17.4)	4 (18.2)	8 (17.8)	
**Vanilla**				0.706
Yes	13 (56.5)	11 (52.4)	24 (54.5)	
No	8 (34.8)	6 (28.6)	14 (31.8)	
Did not test	2 (8.7)	4 (19.0)	6 (13.6)	
**Peanut Butter**				0.416
Yes	17 (73.9%)	19 (86.4)	36 (80.0)	
No	4 (17.4%)	1 (4.5)	5 (11.1)	
Did not test	2 (8.7%)	2 (9.1)	4 (8.9)	

The results of home odorant testing for the patients who completed this are presented here. There was no significant difference in percent of correct answers between the two groups.

Finally, there was poor concordance in our study between techniques used to evaluate olfaction. There were 21 patients with abnormal smell based on SIT, and 20 with abnormal smell based on home testing, but there was poor overlap between the groups (Table 5A). All 4 patients who reported changes in smell and/or test did have abnormal SIT testing, but of the 46 patients who denied subjective changes and completed SIT, only 17 (37%) had normal “normosmia” on SIT (Table 5B).

**Table 5:** Results of SIT testing compared with home odorant-based testing and questionnaire results. **A.** Test results were considered abnormal if patients were diagnosed with any degree of microsmia or anosmia on SIT, or if any question on home-based testing was incorrect. **B.** Test results were considered abnormal if patients were diagnosed with any degree of microsmia or anosmia on SIT, or if they reported “Yes” to questions asking about change in sense of smell or taste in the past 14 days.

A				B			
		Abnormal SIT				Abnormal SIT	
		No	Yes			No	Yes
Abnormal Home Test	No	21	10	Abnormal Questionnaire	No	29	17
	Yes	9	11		Yes	0	4

## Discussion

We hypothesized that a simple version of smell identification testing could be used as a complementary tool in screening for pediatric COVID-19. We found that even with the original SARS-CoV2 variants, subjective and simple home odorant and complex, validated SIT were not useful in discriminating COVID-19 positive children from COVID-19 negative children in the outpatient setting. Unlike previous reports in adults [2, 3, 13, 27], outpatient children with SARS-CoV2 may not experience subjective or measurable changes in olfaction compared to children without COVID-19. This was found even with a sample size that was more than sufficient to detect a significant difference of rate of dysosmia between the 2 groups based on pre-recruitment power calculations. These results differ from recent studies evaluating children and adults hospitalized with COVID-19 [2, 3, 9, 27]. The patients presented here have milder disease than patients in those studies, which could explain the differing results. Our study is the first pediatric study to include an objective assessment of olfactory function, compare COVID-19 positive patients to negative controls and focus on outpatient children diagnosed with COVID-19 on pre-operative or pre-admission testing.

We hypothesized that addition of standardized chemosensory testing would improve identification of olfactory changes that young children would otherwise not be able to describe subjectively. Despite a large majority of all patients reporting no subjective change in smell or taste, relatively few scored within the “normosmia” category on olfactory testing. Evaluating all subjects, only 4 children (6%) answered “yes” to a change in sense of smell. However, only 58.8% of all children included were able to score within the age- and sex-matched “normosmia” category on the University of Pennsylvania Smell Identification Test (UPSIT) (Table 3). And only 39% of all children were able to correctly identify every odorant on home-based testing (Table 5). The high level of olfactory dysfunction noted on SIT in our SARS-CoV2 negative group highlights the challenges of such testing in children. This finding may be because UPSIT is not effective for diagnosing normosmia in children, *or* because children do not accurately report olfactory disruptions when simply asked routine COVID-19 screening questions. This differs from what has been previously been reported in adult outpatients with COVID-19, where subjective reports of hyposmia correlated with scores on SIT [28]. This conclusion underscores the importance of approaching screening algorithms and diagnosis of COVID-19 in children with a uniquely pediatric approach instead of using the same methods used in adults.

Numerous reports in the literature note that odor identification improves with age [20]. This has been attributed to maturation in cognitive, linguistic, and olfactory skills [29]. Using an adult-focused chemosensory protocol may also affect accuracy, (although UPSIT has been validated for children as young as 5 years) [30]. The UPSIT was chosen because it is very broad, one-time-use (important given social distancing considerations), and has been validated in young children. Although certain smells presented may be difficult for young children to identify (i.e. turpentine, whiskey), the SIT scoring system is based on age- and sex-matched normative data in 5 year intervals [16, 20]. Given that similarly low numbers of children were able to accurately identify all home odorants and score within the normosmia category on UPSIT, it is not possible to say whether the low rate of normosmia was related to true olfactory dysfunction even in healthy children or because of the complexity of testing used.

Interestingly, similar findings have been documented even in adults. When asked about sense of smell prior to chemosensory testing, otherwise healthy adults did not accurately report hyposmia based on SIT [17]. Furthermore, in a study of over 9000 participants, 3.4% of adults scored within functionally anosmic categories on SIT, despite reporting that they were otherwise healthy [18]. Taking this into consideration along with developmental challenges when evaluating children, it is not entirely unprecedented that 40% of children scored outside of normosmic categories in the present study.

This study adds to our current understanding of early variants of SARS-CoV2, and how the virus has varying impacts on young children compared to adolescents and adults. Children in our cohort were presumed to not have active upper respiratory infections. In this study, the subjective symptoms reported in the COVID-19 positive group were similar to those in the negative group (Table 2), suggesting that existing screening questionnaires asking about respiratory and chemosensory changes are likely not effective at detecting early COVID-19 in outpatient school children.

The strengths of this study include the prospective design, outpatient population included, inclusion of otherwise healthy controls and the use of multiple forms of olfactory testing in addition to symptoms questionnaires. Additionally, testing was administered within 14 days of diagnosis, the shortest duration of olfactory changes reported in adults [25].

Limitations of this study revolve around sample size, testing and population included. While our study is sufficiently powered to identify differences in normal versus abnormal olfaction, a larger population is required for more specific subgroup analysis. Another limitation is that the UPSIT was used as a comprehensive SIT, not simplified pediatric-specific testing [30] or multi-use pediatric-specific testing [31]. Furthermore, this study represents a homogenous population racially (2/3 of children were White), which limits our ability to understand how these findings may impact people of color. A majority of patients who completed chemosensory testing were recruited prior to emergency use authorization of the COVID-19 vaccines. However, there was 1 vaccinated patient included in the COVID-19-positive cohort. With this very low number, it is not possible to conclude if this confounder affected the overall results.

## Conclusions

In this study, answers to screening questions created based on adult symptoms of COVID-19 were equivalent in pediatric SARS-CoV-2 positive and negative cohorts. Children’s subjective report of chemosensory changes were not inline with scores on UPSIT or home-based odorant testing. There was no difference in SIT scores between COVID-19 pediatric outpatients and controls. The results presented here suggest the need for novel and uniquely pediatric strategies to screen for COVID-19 in outpatient children.

## Supporting information

S1 Data (CSV)

## List of abbreviations

COVID-19: coronavirus disease 2019
SARS CoV2: severe acute respiratory syndrome coronavirus 2
SIT: Smell Identification Test
UPSIT: University of Pennsylvania Smell Identification Test

## References

[1] Hong H, Wang Y, Chung HT, Chen CJ. Clinical characteristics of novel coronavirus disease 2019 (COVID-19) in newborns, infants and children. *Pediatrics & Neonatology*. 2020;61(2):131–132. doi: 10.1016/j.pedneo.2020.03.001 32199864 PMC7129773

[2] Yan CH, Faraji F, Prajapati DP, Boone CE, DeConde AS. Association of chemosensory dysfunction and Covid-19 in patients presenting with influenza-like symptoms. *International Forum of Allergy & Rhinology*. Published online April 12, 2020. doi: 10.1002/alr.22579 32279441 PMC7262089

[3] Moein ST, Hashemian SMR, Mansourafshar B, Khorram‐Tousi A, Tabarsi P, Doty RL. Smell dysfunction: a biomarker for COVID‐19. *International Forum of Allergy & Rhinology*. Published online April 17, 2020:alr.22587. doi: 10.1002/alr.22587 32301284 PMC7262123

[4] Hopkins C, Surda P, Kumar N. Presentation of new onset anosmia during the COVID-19 pandemic. Rhinology. Published online April 11, 2020. doi: 10.4193/Rhin20.116 32277751

[5] COVID-19 Map—Johns Hopkins Coronavirus Resource Center. Accessed May 7, 2020. https://coronavirus.jhu.edu/map.html

[6] Ceccarelli M, Berretta M, Rullo EV, Nunnari G, Cacopardo B. Editorial–Differences and similarities between Severe Acute Respiratory Syndrome (SARS)-CoronaVirus (CoV) and SARS-CoV-2. Would a rose by another name smell as sweet? *European Review for Medical and Pharmacological Sciences*. 2020;24(5):2781–2783. doi: 10.26355/eurrev_202003_20551 32196628

[7] Lai CC, Shih TP, Ko WC, Tang HJ, Hsueh PR. Severe acute respiratory syndrome coronavirus 2 (SARS-CoV-2) and coronavirus disease-2019 (COVID-19): The epidemic and the challenges. *International Journal of Antimicrobial Agents*. 2020;55(3). doi: 10.1016/j.ijantimicag.2020.105924 32081636 PMC7127800

[8] Gorbalenya AE, Baker SC, Baric RS, et al. The species Severe acute respiratory syndrome-related coronavirus: classifying 2019-nCoV and naming it SARS-CoV-2. *Nature Microbiology*. 2020;5(4):536–544. doi: 10.1038/s41564-020-0695-z 32123347 PMC7095448

[9] Rusetsky Y, Meytel I, Mokoyan Z, Fisenko A, Babayan A, Malyavina U. Smell Status in Children Infected with SARS-CoV-2. *Laryngoscope*. Published online 2021. doi: 10.1002/lary.29403 33443298 PMC8013292

[10] Larremore DB, Toomre D, Parker R. Modeling the effectiveness of olfactory testing to limit SARS-CoV-2 transmission. Nature Communications. 2021 Jun 16;12(1):1–9. 10.1038/s41467-021-23315-5 PMC8209051 34135322

[11] Lee B, Raszka W V. COVID-19 in children: Looking forward, not back. *Pediatrics*. 2021;147(1). doi: 10.1542/PEDS.2020-029736 33033179

[12] Dong Y, Mo X, Hu Y, et al. Epidemiology of COVID-19 Among Children in China. *Pediatrics*. Published online March 16, 2020:e20200702. doi: 10.1542/peds.2020-0702 32179660

[13] Bialek S, Gierke R, Hughes M, McNamara LA, Pilishvili T, Skoff T. Coronavirus Disease 2019 in Children—United States, February 12–April 2, 2020. *MMWR Morbidity and Mortality Weekly Report*. 2020;69(14):422–426. doi: 10.15585/mmwr.mm6914e4 32271728 PMC7147903

[14] Ranabothu S, Onteddu S, Nalleballe K, Dandu V, Veerapaneni K, Veerapandiyan A. Spectrum of COVID-19 in children. *Acta Paediatrica*, *International Journal of Paediatrics*. 2020;109(9):1899–1900. doi: 10.1111/apa.15412 32538518 PMC7323213

[15] Mak PQ, Chung KS, Wong JSC, Shek CC, Kwan MYW. Anosmia and ageusia: Not an uncommon presentation of COVID-19 infection in children and adolescents. *Pediatric Infectious Disease Journal*. 2020;39(8):E199–E200. doi: 10.1097/INF.0000000000002718 32516281

[16] Hugh SC, Siu J, Hummel T, et al. Olfactory testing in children using objective tools: Comparison of Sniffin’ Sticks and University of Pennsylvania Smell Identification Test (UPSIT). *Journal of Otolaryngology—Head and Neck Surgery*. 2015;44(March). doi: 10.1186/s40463-015-0061-y 25890082 PMC4359791

[17] Landis BN, Hummel T, Hugentobler M, Giger R, Lacroix JS. Ratings of overall olfactory function. Chemical senses. 2003 Oct 1;28(8):691–4. doi: 10.1093/chemse/bjg061 14627537

[18] Oleszkiewicz A, Hummel T. Whose nose does not know? Demographical characterization of people unaware of anosmia. European Archives of Oto-Rhino-Laryngology. 2019 Jun;276(6):1849–52. doi: 10.1007/s00405-019-05414-8 30989334 PMC6529373

[19] Landis BN, Hummel T. Measuring olfaction instead of asking: it is more than luxury! European Archives of Oto-Rhino-Laryngology. 2020 Jun;277(6):1843–4. 31325033 10.1007/s00405-019-05565-8

[20] Doty RL, Shaman P, Kimmelman CP, Dann MS. University of Pennsylvania smell identification test: A rapid quantitative olfactory function test for the clinic. *Laryngoscope*. 1984;94(2):176–178. doi: 10.1288/00005537-198402000-00004 6694486

[21] SmellTracker. Accessed April 2, 2020. http://www.smelltracker.org/

[22] Harris PA, Taylor R, Thielke R, Payne J, Gonzalez N, Conde JG. Research electronic data capture (REDCap)—a metadata-driven methodology and workflow process for providing translational research informatics support. *Journal of biomedical informatics*. 2009;42(2):377–381. doi: 10.1016/j.jbi.2008.08.010 18929686 PMC2700030

[23] Landis BN, Konnerth CG, Hummel T. *A Study on the Frequency of Olfactory Dysfunction*.; 2004. 10.1097/00005537-200410000-00017 15454769

[24] Nordin S, Brämerson A. Complaints of olfactory disorders: Epidemiology, assessment and clinical implications. *Current Opinion in Allergy and Clinical Immunology*. 2008;8(1):10–15. doi: 10.1097/ACI.0b013e3282f3f473 18188011

[25] Hopkins C, Surda P, Whitehead E, Kumar BN. Early recovery following new onset anosmia during the COVID-19 pandemic—an observational cohort study. *Journal of otolaryngology—head & neck surgery*. 2020;49(1):26. doi: 10.1186/s40463-020-00423-8 32366299 PMC7196882

[26] Speth MM, Singer-Cornelius T, Obere M, Brockmeier SJ, Sedaghat AR. *Olfactory Dysfunction and Sinonasal Symptomatology in COVID-19*: *2 Prevalence*, *Severity*, *Timing and Associated Characteristics 3 4 Introduction 75*. 10.1177/0194599820929185 PMC7240313 32423357

[27] Vaira LA, Deiana G, Fois AG, et al. Objective evaluation of anosmia and ageusia in COVID-19 patients: Single-center experience on 72 cases. In: *Head and Neck*. Vol 42. John Wiley and Sons Inc.; 2020:1252–1258. doi: 10.1002/hed.26204 32342566 PMC7267244

[28] Prajapati DP, Shahrvini B, MacDonald BV, et al. Association of subjective olfactory dysfunction and 12‐item odor identification testing in ambulatory COVID‐19 patients. *International Forum of Allergy & Rhinology*. 2020;10(11). doi: 10.1002/alr.22688 32964657

[29] Cameron EL. Olfactory perception in children. *World journal of otorhinolaryngology-head and neck surgery*. 2018;4(1):57–66. doi: 10.1016/j.wjorl.2018.02.002 30035263 PMC6051253

[30] Cameron EL, Doty RL. Odor identification testing in children and young adults using the smell wheel. *International journal of pediatric otorhinolaryngology*. 2013;77(3):346–350. doi: 10.1016/j.ijporl.2012.11.022 23246420

[31] Schriever VA, Mori E, Petters W, Boerner C, Smitka M, Hummel T. The “Sniffin’Kids” test-a 14-item odor identification test for children. PLoS One. 2014 Jun 30;9(6):e101086. doi: 10.1371/journal.pone.0101086 24979650 PMC4076236

